# Dual challenge inside the womb: a case report of concomitant fetal atrio-ventricular block associated with maternal anti-SSA antibodies and fetal tachyarrhythmia diagnosed as Wolff-Parkinson-White syndrome after birth

**DOI:** 10.3389/fimmu.2024.1397103

**Published:** 2024-07-24

**Authors:** Ana Teodósio Chícharo, Mónica Rebelo, Ana Rita Lopes, Maria João Saavedra, Maria Filipa Paramés, Ana Rita Araújo, Ana Rita Cruz-Machado, Luísa Pinto, Susana Capela

**Affiliations:** ^1^ Rheumatology Department, Unidade Local de Saúde do Hospital de Santa Maria, Lisbon, Portugal; ^2^ Rheumatology Department, Unidade Local de Saúde do Algarve, Hospital de Faro, Faro, Portugal; ^3^ Department of Pediatric Cardiology, Unidade Local de Saúde do Hospital de Santa Maria, Lisbon, Portugal; ^4^ Instituto de Medicina Molecular João Lobo Antunes, Faculdade de Medicina da Universidade de Lisboa, Lisbon, Portugal; ^5^ Department of Clinical Pathology, Unidade Local de Saúde do Hospital de Santa Maria, Lisbon, Portugal; ^6^ Department of Obstetrics, Gynecology and Reproductive Medicine, Unidade Local de Saúde do Hospital de Santa Maria, Lisbon, Portugal

**Keywords:** case report, fetal atrio-ventricular block, congenital heart block, Wolff-Parkinson-White syndrome, pregnancy, anti-SSA/Ro antibodies

## Abstract

Fetal autoimmune atrioventricular block (AVB) is a rare but potentially life-threatening condition. It results from the passage of maternal anti-SSA/Ro or Anti SSB/La auto-antibodies into the fetal circulation, leading to inflammation and fibrosis of the AV node and often to irreversible damage. Besides AVB, these antibodies can also cause cardiomyopathies, but there is no evidence linking them to tachyarrhythmias. We present the case of a patient with significant risk factors for fetal AVB: a prior history of *hydrops fetalis*, high anti-SSA/Ro antibody levels and hypothyroidism. In this case, the use of dexamethasone and intravenous immunoglobulin may have contributed to reversing the first-degree atrioventricular block detected at 19 weeks of gestation. Additionally, at 21 weeks, the fetus developed a tachyarrhythmia that needed treatment with flecainide. Soon after the birth, the newborn underwent ECG Holter and Wolff-Parkinson-White Syndrome (WPWS) was diagnosed. To our knowledge, the coexistence of fetal AVB and WPWS has never been described.

## Introduction

Fetal autoimmune AVB is amongst the manifestations collectively referred to as neonatal lupus. Fetal AVB results from the passage of maternal anti-SSA/Ro and/or anti-SSB/La auto-antibodies into the fetal circulation, leading to inflammation and fibrosis of the atrioventricular node, often causing irreversible damage. Fetal AVB occurs in 2–5% of pregnancies in women with anti-SSA/Ro and/or anti-La/SSB antibodies, but the rate in subsequent gestations may be as high as 17% (1). Thyroid pathology also increases the risk of fetal AVB in mothers with anti-SSA/Ro antibodies. On the other hand, WPWS is a congenital heart condition that occurs in 0.1% of neonates (2). It is characterized by an abnormal electrical pathway between the atria and ventricles that leads to tachycardia. To the best of our knowledge, there is only one other case in the literature describing WPWS in a newborn with neonatal lupus (3). Herein, we present a unique case of coexistence of immune AVB and WPWS in the offspring of a woman with anti-SSA/Ro antibodies, underlining the diagnostic and therapeutic challenges faced throughout pregnancy.

## Case description

We present the case of a 29-year-old Caucasian female with hypothyroidism, medicated and controlled with levothyroxine 37.5 µg/day. She had a normal body mass index (20.4 kg/m^2^) and denied toxic habits. Her family history was irrelevant. She denied previous surgeries and other physical or mental issues. She got pregnant for the first time in the beginning of 2022. This pregnancy, desired and planned, was uneventful until 21 weeks of gestation (WG), when the routine obstetric ultrasound identified a severe *hydrops fetalis*. The fetal echocardiogram revealed a third-degree AVB (atrial heart rate 140 bpm, ventricular heart rate 47 bpm). Initial laboratory results showed positive antinuclear antibodies (titer 1:1280) with strongly positive anti-SSA/SSB antibodies. At this point, the couple was informed of the poor prognosis of the situation and medical termination of pregnancy was performed at 23 WG.

At this time, the patient’s concerns were tied to her desire to become a mother and the fear of the recurrence of this situation. She was subsequently referred to preconceptional counseling at our rheumatology/obstetrics multidisciplinary clinic in a tertiary hospital, that took place in July of 2022.

A comprehensive medical history revealed that the patient had been experiencing chilblains, fatigue and sporadic mechanical arthralgia over the past two years. She denied symptoms such as *sicca* syndrome, oral ulcers, alopecia, skin lesions, *Raynaud* phenomenon, fever, weight loss or any other symptoms of connective tissue disease. Physical examination yielded unremarkable findings. A more detailed laboratory work-up revealed high anti-SSA/Ro titers. Anti-dsDNA and antiphospholipid antibodies were negative. Inflammatory parameters (erythrocyte sedimentation rate and C-reactive protein), complement levels (C3 and C4 levels) and thyroid function were normal. No significant changes were detected through capillaroscopy, salivary gland ultrasound or biopsy. Echocardiogram and thoracic CT also showed no remarkable findings.

Given her intention to conceive again, preconception counseling was provided, which involved a discussion about the increased risk of AVB in the subsequent gestation (up to 17% according to international series) (1). She was advised to seek care at a high-risk pregnancy clinic, with multidisciplinary appointments and a recommendation to undergo weekly fetal echocardiograms starting at 16 WG. Additionally, she was prescribed hydroxychloroquine at a dose of 400mg/day.

Six months later, the patient became pregnant again and received care at our obstetric-rheumatology unit. She reported no symptoms. The routine first trimester ultrasound showed no abnormalities. The risk of preeclampsia, assessed by a score that includes clinical data, blood pressure, Doppler of the uterine arteries and laboratory results [pregnancy-associated plasma protein-A (PAPP-A) and placental growth factor (PlGF)], revealed a low risk. Anti-SSA/Ro titers remained high [anti-Ro52 4236 UQ CU (chemiluminescent units); anti-Ro60 >27496 UQ CU], while anti-SSB/La was slightly above the normal range (7.4 UQ; cut-off 5 UQ). As programmed, weekly fetal echocardiograms were initiated at 16 WG.

**Figure 1:**
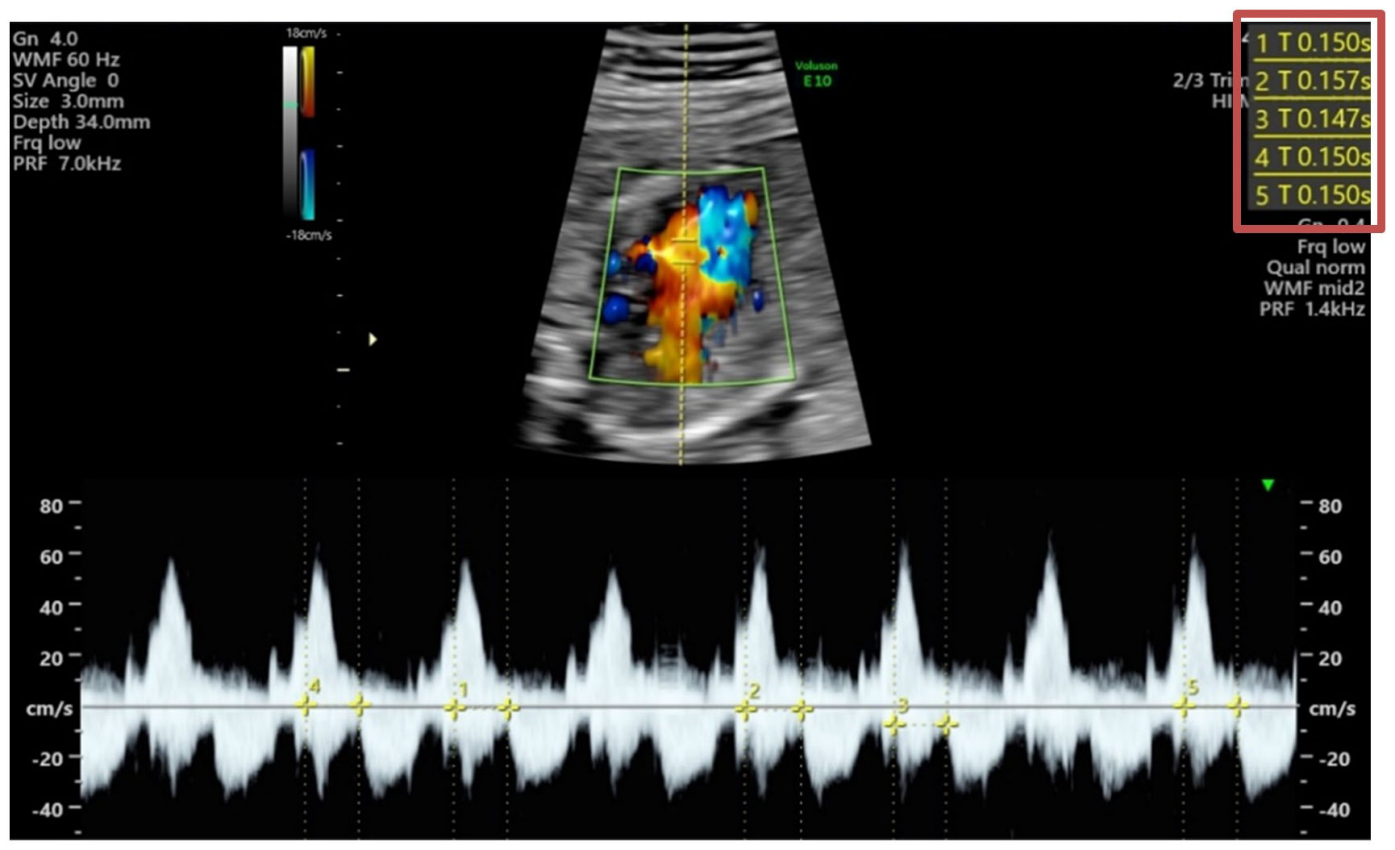
Fetal echocardiogram (2D image with color Doppler and flow Doppler) at 19 weeks of gestation, showing a prolongation of mechanical PR interval. On the top right corner of the image are presented the different measurements: 150, 157, 147, 150, 150 milliseconds.

At 19 WG, the patient reported extreme fatigue, without dyspnea, thoracic pain or other symptoms. Two consecutive fetal echocardiograms revealed a prolonged mechanical PR interval (150-157 msec), suggestive of a first-degree AVB (**Figure 1**), with no structural or functional heart disease. Given her high-risk profile (previous *hydrops fetalis*, hypothyroidism and high levels of anti-SSA/Ro antibodies), it was decided to initiate dexamethasone 8 mg/day (together with calcium and vitamin D3 supplementation) and intravenous immunoglobulin (IVIG) at a dose of 1 g/Kg (4). The Nephrology team was consulted at this point to consider plasmapheresis if the heart block progressed to second degree. Fortunately, four days later, the first-degree fetal AVB had reversed (PR interval 116-130 msec) and anti-SSA antibody titers had decreased [anti-Ro52 1232 FLU (fluorescence units); anti-Ro60 15647 FLU]. For the first time, complement consumption was noted (C4 4 mg/dl), probably related to the IVIG therapy, as the patient dramatically improved her fatigue (5). The IVIG perfusions were maintained twice a week and dexamethasone was gradually tapered down to 4 mg/day.

**Figure 2:**
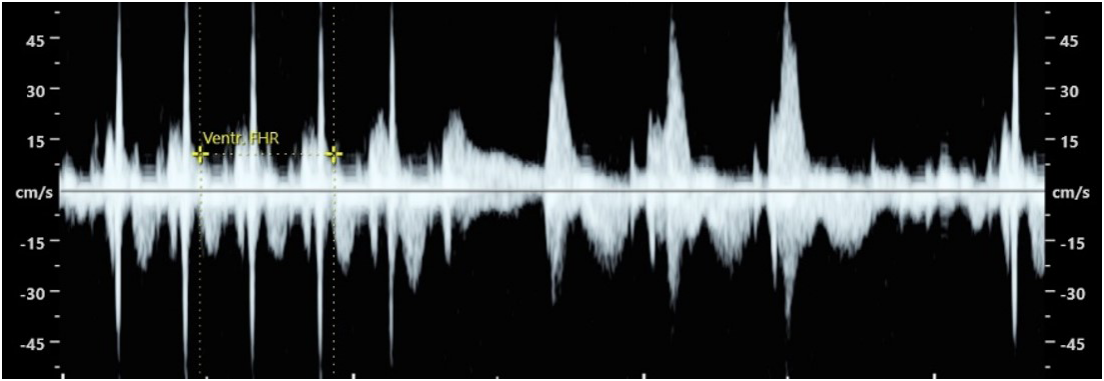
Fetal echocardiogram (Flow Doppler) at 21 weeks of gestation, revealing fetal supraventricular tachycardia alternating with sinus rhythm (a more explicit video is presented in **Supplementary Material**).

At 21 WG, a fetal echocardiogram revealed short periods of paroxysmal supraventricular tachycardia (fetal heart rate 260 bpm; normal PR interval 124-129 msec) (**Figure 2**). The patient was initially advised with general measures, such as ensuring adequate hydration, and avoiding stimulants and anxiety-inducing situations. However, at 22 WG, a subsequent fetal echocardiogram showed that the fetus was in supraventricular tachycardia, with only small periods in sinus rhythm, and a small pericardial effusion was noted. Flecainide at a dose of 100 mg every 8 hours was introduced at this point. Despite being the safest antiarrhythmic choice regarding the risk of bradycardia, the patient and the fetus remained under close monitoring. After 3 weeks (6 perfusions) of IVIG, this therapy was discontinued, as tachyarrhythmias in children potentially triggered by this drug were reported in the literature (6, 7). Complement levels of C4 remained low throughout the IVIG therapy (from 19-21 WG) and returned to normal range in the beginning of third trimester. Dexamethasone was stopped at 28 WG, considering serial normal fetal echocardiograms. Flecainide was continued throughout pregnancy with dose adjustments based on serum drug levels.

At 37 WG, the patient reported extreme fatigue and antibody titers increased once again (anti-Ro52 695 FLU; anti-Ro60 8498 FLU). Due to the similarity of the clinical presentation to what occurred at 19 WG, it was decided to repeat IVIG infusion before delivery. This also aimed to enhance immunomodulation in the newborn. Once again, the extreme fatigue resolved with this medication.

**Figure 3:**
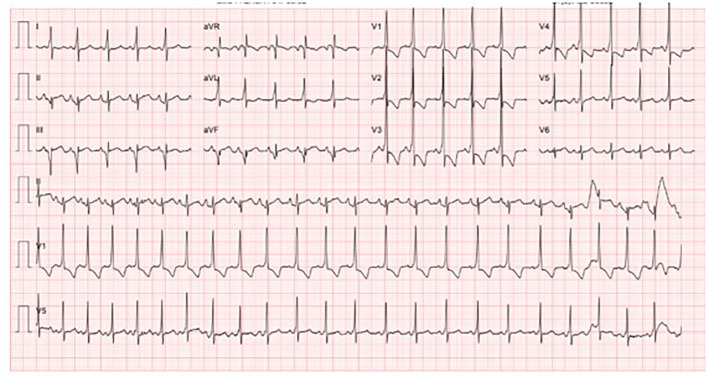
Holter of the newborn at 1 month-old. It. It is noticeable a short PR interval and a slurred QRS complex (delta wave) compatible with a Wolf-Parkinson-White Syndrome.

At 38 WG, she went into labor after spontaneous rupture of membranes and a vacuum extraction was performed. A healthy female baby weighing 2490g (percentile 5.2) was bornshowing no rash, cytopenia, hepatic dysfunction or any other manifestation of neonatal lupus. The neonate was started on propranolol due to the previous *in utero* tachyarrhythmia. At 1 month-old, a Holter monitoring revealed a short PR interval and a slurred QRS complex, consistent with WPWS (**Figure 3**).

**Figure 4:**
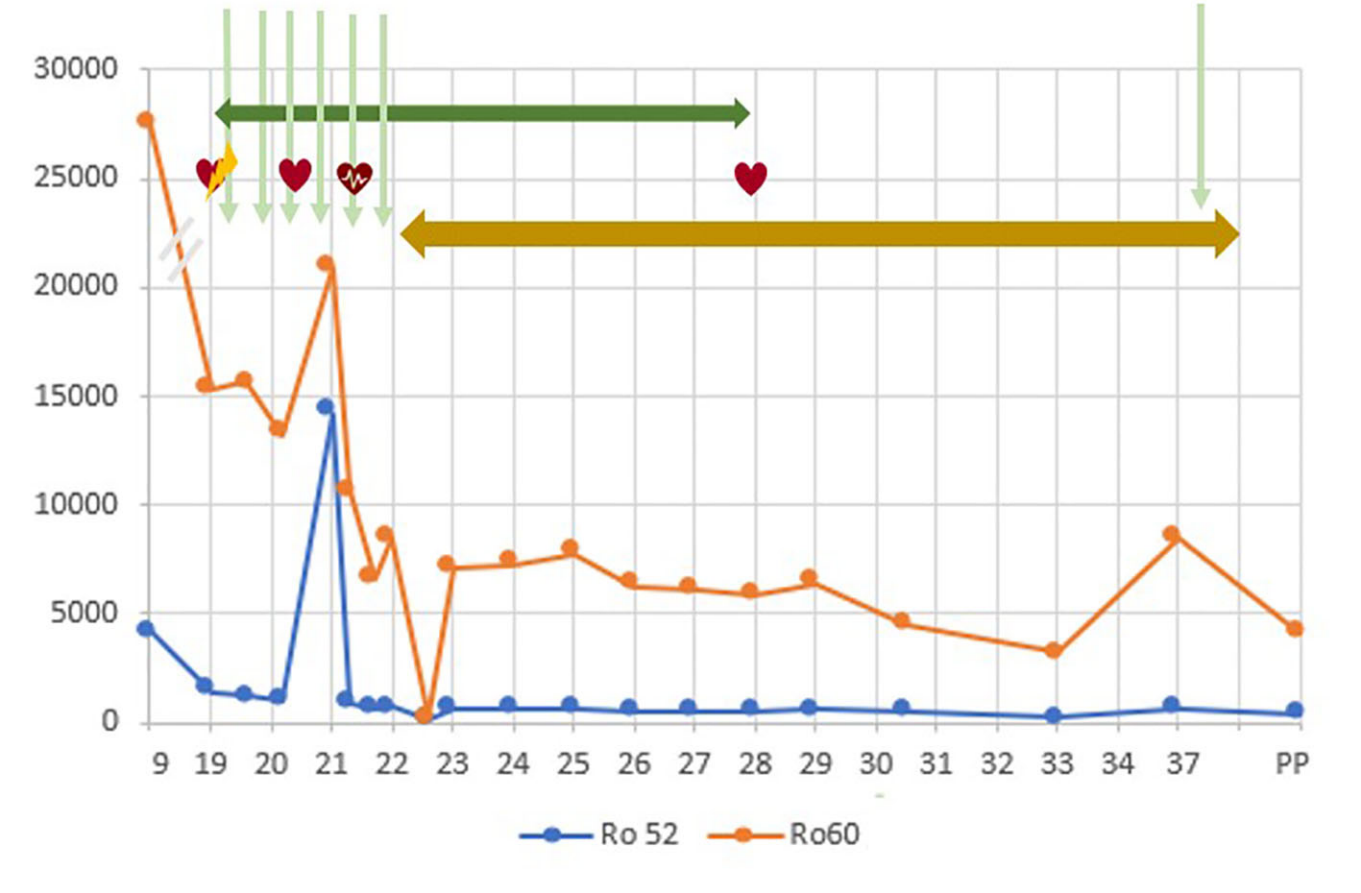
Timeline of events, from the beginning of pregnancy until the postpartum period. In X axis, the weeks of gestation. In Y axis, units of anti-SSA/Ro antibodies (at 9 WG, in UQ; from 19 WG on in FLU units). The blue line represents the Ro52 levels and the orange line represents the Ro60 levels. PP, postpartum 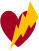
First degree AV block 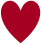
Sinus rhythm 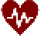
Supraventricular tachycardia 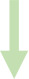
Intravenous immunoglobulin 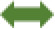
Dexamethasone 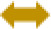
Flecainide

In June of 2024, the baby is now 10 months old and exhibits normal psychomotor development. The child is still under antiarrhythmic treatment, which will be maintained until she reaches one year of age. Regular follow-up appointments with pediatric cardiology are ongoing, and no new episodes of rhythm alteration were identified so far. **Figure 4** illustrates the timeline of events, from the beginning of pregnancy until the postpartum period.

## Discussion

Neonatal lupus erythematosus (NLE) encompasses several fetal and child manifestations associated with maternal anti-Ro/SSA and, less frequently, anti-La/SSB auto-antibodies. This condition may affect children born to mothers with a pre-existing connective tissue disorder, primarily Sjogren’s syndrome or systemic lupus erythematosus. However, most cases are documented in asymptomatic antibody carriers (8). Manifestations linked to these antibodies include rash (in approximately 10%), transient cytopenia (around 20%) and mild transaminases elevation (approximately 30%). These complications resolve spontaneously as maternal antibodies disappear from the baby´s circulation (9). The most concerning manifestation associated with these antibodies is autoimmune congenital heart block, which may result in irreversible fetal complications and death. Anti-SSA/SSB antibodies can cross the placental barrier as early as week 11 of gestation, causing damage to fetal conduction tissues through inflammation, calcification and fibrosis, ultimately blocking signal conduction at the AV node (10).

To prevent AVB, the 2020 American College of Rheumatology Guidelines for the Management of Reproductive Health in Rheumatic and Musculoskeletal Diseases recommends hydroxychloroquine during pregnancy for all women with anti-SSA/Ro and/or anti-SSB/La antibodies (10). Studies have indicated that exposure to hydroxychloroquine during fetal development may reduce the recurrence of congenital heart block by more than 50% (11, 12). This protective effect is attributed to hydroxychloroquine’s ability to inhibit Toll-like receptor 7 stimulation, preventing a cascade of events that lead to the secretion of pro-inflammatory substances and subsequent cardiac fibrosis (13, 14). Despite limited data, the safety profile of hydroxychloroquine makes it a favorable option.

Pregnant women with positive anti-SSA/Ro antibodies and a history of a previous infant with congenital heart block or other NLE manifestations should be referred for fetal heart evaluation by a pediatric cardiologist (10). Fetal cardiac conduction intervals, particularly the AV interval, should be closely monitored. American guidelines for the management of reproductive health in rheumatic diseases recommend weekly fetal echocardiography starting at weeks 16–18 and continuing up to week 26 in these high-risk patients with a previous fetal AVB (15).

Fetal AVB is considered a dynamic condition where lower-degree AVB can spontaneously revert or, on the other hand, progress to a higher-degree AVB within 24 hours (15). Treatment of fetal first-degree AVB is controversial but, recognizing that once third-degree AVB is installed the damage is irreversible, it is still recommended by international guidelines (10).

Regarding complete or third-degree AVB, no therapy has shown to improve mortality and morbidity rates associated with this condition. However, concerning incomplete AVB, the optimal pharmacological approach remains a matter of debate and an individual choice of the multidisciplinary clinic, based on local experience and available treatments. Various therapeutic interventions have been reported, mostly in single cases or small series, including corticosteroids, IVIG, plasmapheresis, beta-adrenergic agents, B cell depletion therapies, immunosuppressive agents and hydroxychloroquine (8).

According to American College of Rheumatology Guidelines for the Management of Reproductive Health in Rheumatic and Musculoskeletal Diseases, if first or second-degree heart block are detected in a fetal echocardiogram, a brief course of dexamethasone is recommended (15). As a fluorinated steroid, dexamethasone can cross the placenta, exerting its effects on the developing fetus (8). Considering corticosteroids have anti-inflammatory effects, maternal administration of dexamethasone might prevent fetal AVB progression to a higher degree by mitigating the inflammatory response and potentially preventing the establishment of fibrosis of the conduction system. There may be a potential “window of opportunity” for treatment, before fibrosis is installed. Nevertheless, there are concerns about prolonged fetal exposure to corticosteroids, as it may lead to fetal growth restriction, oligohydramnios and potential long-term neurodevelopmental effects (16).

Regarding treatment with IVIG, the evidence is controversial and there is no standardized regimen. Some studies have concluded that IVIG in a dose of 400 mg/kg was ineffective as prophylactic therapy for preventing fetal AVB in high-risk pregnancies (17, 18). However, contrasting evidence suggests that IVIG may prevent the progression of immune-mediated fetal AVB by decreasing maternal serum antibody levels and potentially inhibiting the transfer of pathogenic anti-Ro/La antibodies across the placenta (19). This hypothesis is supported by murine model studies, case reports and small series using IVIG at a dose of 1mg/kg at different gestation timings (4, 20–22).

Our case report describes a patient with three significant risk factors for fetal AVB: a prior history of *hydrops fetalis*, high anti-SSA/Ro antibody levels and hypothyroidism. In a subsequent pregnancy, under hydroxychloroquine, a first degree AVB [PR interval of ≥150 msec (21)] was detected in a routine fetal echocardiogram at 19 WG, during an episode of extreme fatigue of the mother. At this point, the additional use of dexamethasone and IVIG might have contributed to revert the AVB, as there was a gradual reduction of maternal antibody titers and improvement in maternal symptoms. The unexpected turn of events occurred with the onset of fetal tachyarrhythmia starting at 21 WG, leading to the discontinuation of IVIG, due to concerns about its potential iatrogenic effect, and to the introduction of flecainide. In this case, starting antiarrhythmic therapy was a crucial but difficult decision, due to the potential of eliciting an AV block, a feared complication since the beginning of pregnancy. Only after birth the cause of the fetal tachyarrhythmia was identified: a concomitant WPWS diagnosis in the newborn detected by Holter.

As far as we are aware, there is only one other manuscript describing a case of NLE concomitant with WPWS. This Thai paper describes a case series of 6 newborns with NLE, all exhibiting cutaneous manifestations. Among them, one newborn had abnormal electrocardiogram changes compatible with WPWS, while no heart blocks were described (3).

Uncertainty remains regarding whether this occurrence of WPWS could potentially be another manifestation of the presence of anti-SSA/Ro antibodies, or merely a coincidental event with significant therapeutic implications.

This case highlights the importance of a multidisciplinary approach in evaluating and surveilling pregnant women with anti-SSA/SSB antibodies before, during, and after pregnancy. Prenatal management holds crucial clinical implications regarding fetal prognosis. Early detection of a fetal AVB can provide a “window of opportunity” to reverse the situation. Prompt intervention in an incomplete AVB can optimize fetal-maternal outcomes and mitigate the risks associated with autoimmune-mediated fetal AVB. However, there is a need for further research to establish clear protocols for the management of incomplete heart block in fetuses of mothers with positive anti-SSA/SSB antibodies. The additional diagnosis of WPWS in the newborn increased the complexity of this case.

## Patient perspective

“At 19 weeks of gestation, during my fourth fetal echocardiogram, a first-degree AV block was detected. This was my biggest fear, due to my SSA antibodies and past experience. Later in pregnancy, fetal tachyarrhythmia was also identified. The medical team (rheumatology, cardiology, and obstetrics) overseeing my care acted promptly to reverse the situation and stabilize the baby’s cardiac health, always explaining all procedures and their possible side effects. I remained well throughout the treatments, diligently following all medical advice, hoping for a happy outcome. Each treatment was accompanied by some anxiety, fearing that the SSA antibody levels would rise again and affect the baby. However, with the support of my family and the medical team, I felt confident and secure that this situation represented a challenge we would overcome, and that we would have our baby in our arms. Every week was a victory for all of us. At 38 weeks, Mara decided to be born beautiful and healthy”.

## Data availability statement

The original contributions presented in the study are included in the article/**Supplementary Material**. Further inquiries can be directed to the corresponding author.

## Ethics statement

Written informed consent was obtained from the individual(s) for the publication of any potentially identifiable images or data included in this article.

## Author contributions

AC: Writing – original draft, Writing – review & editing. MR: Writing – review & editing. AL: Writing – review & editing. MS: Writing – review & editing. MP: Writing – review & editing. AA: Writing – review & editing. AC: Writing – review & editing. LP: Writing – review & editing. SC: Writing – review & editing.

## Supplementary material

Supplementary Material We attach a short video of the fetal echocardiogram at 21 weeks of gestation, showing fetal supraventricular tachycardia alternating with sinus rhythm.
