# Supplementary material for: Dual challenge inside the womb: a case report of concomitant fetal atrio-ventricular block associated with maternal anti-SSA antibodies and fetal tachyarrhythmia diagnosed as Wolff-Parkinson-White syndrome after birth
Source: Front Immunol. 2024 Jul 24;15:1397103. doi: 10.3389/fimmu.2024.1397103 (PMC11303133; doi:10.3389/fimmu.2024.1397103)
Supplement: Supplementary Material — We attach a short video of the fetal echocardiogram at 21 weeks of gestation, showing fetal supraventricular tachycardia alternating with sinus rhythm. [file Presentation_1.pptx]

## Slide 1
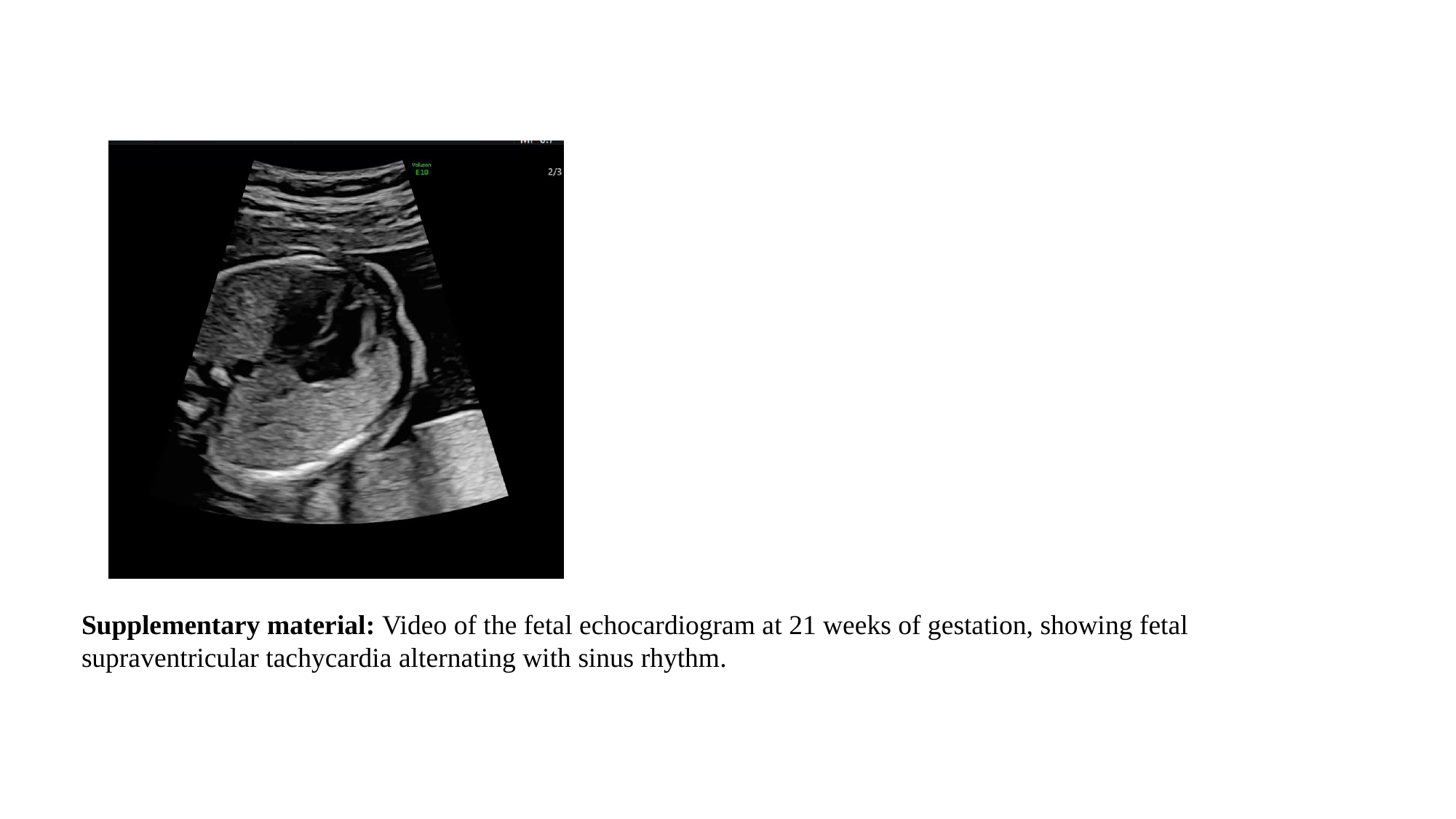

Supplementary material: Video of the fetal echocardiogram at 21 weeks of gestation, showing fetal supraventricular tachycardia alternating with sinus rhythm.
